# The views and experiences of people with type 2 diabetes being cared for by their community pharmacist: a cross-sectional patient survey

**DOI:** 10.1007/s40200-022-01111-2

**Published:** 2022-08-19

**Authors:** Philip Cooney, Jessie Hanley, Nicola Ryan-O’Brien, Hiroshi Okada, Margaret Bermingham

**Affiliations:** 1 Pharmaceutical Care Research Group, School of Pharmacy, University College Cork, Pharmacy Building, College Road, Cork, Ireland; 2 Department of Health Informatics, Graduate School of Medicine & School of Public Health, Yoshida-Konoe-cho, Sakyo-ku, Kyoto, 606-8501 Japan; 3 Division of Preventive Medicine, Clinical Research Institute, National Hospital Organization, Kyoto Medical Center, 1-1 Fukakusa-mukaihata-cho, Fushimi-ku, Kyoto, 612-8555 Japan

**Keywords:** Community pharmacy, Pharmacist, Type 2 diabetes, Ireland, Questionnaire

## Abstract

**Purpose:**

Community pharmacists are highly accessible healthcare providers and studies in several countries have demonstrated a role for community pharmacists in delivering enhanced care to people with type 2 diabetes. The aim of this study is to evaluate the views and experiences of people with type 2 diabetes attending community pharmacies in Ireland.

**Methods:**

A 13-item questionnaire, anchored on a 5-point Likert scale, was used. The study took place in seven pharmacies in the Munster region of Ireland. Participants were people attending a participating pharmacy who had type 2 diabetes and were aged ≥ 18 years.

**Results:**

The questionnaire was answered by 125 people with type 2 diabetes. Mean age of participants was 65.7 ± 12.4 years and 59.2% were male. The statement “I am totally satisfied with my visit to this pharmacist”, was the item that participants most frequently agreed with (agree/strongly agree = 99.2%, mean score 4.9 ± 0.4). Over 80% of participants agreed or strongly agreed with the statement “It is easier to get to see the pharmacist than the doctor”, (mean score 4.3 ± 1.1).

**Conclusion:**

In this population, people with type 2 diabetes were highly satisfied with the care provided to them by their community pharmacist. These data support the implementation of enhanced community pharmacy services for people with type 2 diabetes in Ireland.

## Introduction

Internationally, the incidence and prevalence of type 2 diabetes is increasing [1]. There are approximately 139,000 people with diabetes in Ireland, 90% of whom have type 2 diabetes [2, 3]. At present in Ireland, pharmacies receive reimbursement for dispensing medicines and for certain clinical activities such as vaccine administration and supply of emergency hormonal contraception. Pharmacy services not reimbursed include medicines use reviews, health screening and chronic disease management [4].

The Future of Pharmacy in Ireland report recommends introducing pharmacy-based clinical services for people with chronic diseases including diabetes [5]. This proposal is supported by evidence from several countries, including Canada [6–8], Australia [9] and the United Kingdom (UK) [10] demonstrating the clinical benefits associated with community pharmacist interventions for people with type 2 diabetes. In Canada, studies of extended pharmacist practice in diabetes care have employed interventions such as pharmacist-delivered education and counselling [6–8] as well as pharmacist prescribing [6, 7]. In the SCRIP-HTN study, participants in the pharmacist intervention arm had significantly lower blood pressure than those receiving usual care [8]. In the RxING study, after a six-month pharmacist prescribing intervention, a significant mean reduction in HbA1c and fasting plasma glucose was observed, with half of participants achieving their target HbA1c [7]. Furthermore, in the RxEACH study, participants in the intervention arm receiving a complex intervention that included pharmacist prescribing, experienced a significant reduction in HbA1c, blood pressure, LDL-cholesterol and tobacco use [6].

In Australia and the UK, studies have described complex pharmacist-led interventions in people with type 2 diabetes [9, 10]. Both Krass et al. [9] and Ali et al. [10] provided diabetes education to pharmacists prior to delivery of a pharmaceutical care intervention. In the study conducted in Australia by Krass et al*.*, participants undertook blood glucose monitoring at least once daily and an electronic record of their glycaemic control was reviewed at each of five visits to the pharmacist over a six-month period [9]. At each visit, the glycaemic control record was used to as the basis for discussion and counselling and to identify further interventions such as medication adherence support, identification of drug-related problems, and referral to general practitioner where appropriate [9]. The intervention demonstrated a significant reduction in participants’ HbA1c compared to the control arm over the six-month study period [9]. Ali and colleagues conducted a study in the UK, where participants received six pharmacy appointments over a 12-month period [10]. Diabetes indicators reviewed during the study were body mass index, blood pressure, blood glucose, HbA1c and blood lipids. Based on these indicators, pharmacists provided medication use review, lifestyle counselling and referral to GP as appropriate [10]. Participants in the intervention arm had significantly reduced systolic blood pressure, blood glucose and HbA1c compared with the control group after the 12-month study period [10].

A systematic review concluded that community pharmacy health promotion interventions may benefit health‐related behaviour, intermediate clinical outcomes such as reduction in HbA1c and cholesterol levels, and quality of life for people with chronic disease, including diabetes [11] Furthermore, a review by Collins et al. demonstrated that pharmacist interventions are associated with a significant improvement in glycaemic control among people with diabetes [12]. The review by Collins et al. included 14 studies reporting pharmacist interventions, many of which used complex interventions [12]. Elements included in these complex interventions included education or counselling on diabetes, medication regimen, potential complications of diabetes, diet, and exercise [12]. Other reported elements of the complex pharmacist interventions included blood glucose monitoring, drug therapy monitoring and adjustment of antihyperglycaemic medication, assessment of medication adherence, self-management support, and development of individual care plans [12]. Qualitative studies have shown that such pharmacist interventions are welcomed by people with diabetes [13, 14].

## Aim

There are no Irish reports on the acceptability of pharmacist delivered care for people with type 2 diabetes. Therefore, this study aims to evaluate the views and experiences of people with type 2 diabetes attending community pharmacies in Ireland.

## Ethics approval

The study was approved by the Clinical Research Ethics Committee of the Cork Teaching Hospitals, approval reference ECM 3 (w) 07/03/18. All participants included in the study gave informed consent.

## Method

A cross-sectional survey was conducted in seven pharmacies in January and February 2018. The questionnaire was administered in each location by a final year pharmacy student from the School of Pharmacy, University College Cork, Ireland. All pharmacies were located in the Munster region; one in an urban area, three in suburban areas, and three in a provincial town. The questionnaire was administered in person, on a paper-based form, in the consultation room of each study pharmacy.

The questionnaire was adapted by the authors (HO, MB) from Stewart et al*.* [15] The original questionnaire was used to measure patient acceptability of pharmacist prescribing. This service is not available in Ireland and so questions directly related to prescribing were removed from the instrument. Furthermore, the expression “pharmacist prescriber” was amended to “pharmacist” in the modified questionnaire. The questionnaire consisted of a single section with 13 statements, each scored on a five-point Likert Scale anchored by strongly disagree and strongly agree. All statements concerned the participant’s visit to the pharmacist and the participant’s professional relationship with the pharmacist. The full list of questionnaire statements is given in Table 1. In addition, patient age, sex and diabetes-related medicines were recorded.

**Table 1:** Mean score for each survey statement, presented as mean score ± standard deviation

Statement number	Statements	Statement Mean Score (± standard deviation)
1	I am totally satisfied with my visit to this pharmacist	4.9 ± 0.4
2	This pharmacist told me everything about my treatment	4.7 ± 0.6
3	Some things about my consultation with the pharmacist could have been better	1.7 ± 1.1
4	This pharmacist was interested in me as a person, not just my illness	4.6 ± 0.8
5	I understand my illness much better after seeing this pharmacist	4.5 ± 0.8
6	I felt this pharmacist really knew what I was thinking	4.3 ± 0.9
7	I wish it had been possible to spend a little more time with the pharmacist	2.7 ± 1.4
8	I would find it difficult to tell this pharmacist about some private things	1.9 ± 1.3
9	It is easier to get to see the pharmacist than the doctor	4.3 ± 1.1
10	I get more time with the pharmacist than my doctor(s) for discussing my health-related issues	3.5 ± 1.4
11	I am more comfortable discussing medication-related issues with the pharmacist than my doctor	3.5 ± 1.2
12	I am more interested in the quality of care than the profession of the person who provides it	4.2 ± 1.1
13	I would recommend seeing a pharmacist to other people	4.7 ± 0.7

Study participants were people with type 2 diabetes, who were aged 18 years and older and were attending a participating community pharmacy with a prescription for an antihyperglycaemic medication, insulin or blood glucose test strips. All eligible patients who attended a participating pharmacy during the study period and who fulfilled the inclusion criteria were invited to participate in the study. No distinction was made between people with type 2 diabetes who regularly attended a study pharmacy and those who were new patients to the pharmacy.

A score was allocated to each Likert scale response, with strongly agree = 5, agree = 4, neutral = 3, disagree = 2 and strongly disagree = 1. The mean score (± standard deviation) of each questionnaire statement was then calculated. The percentage of participants answering “agree” or “strongly agree” and the percentage of participants answering “disagree” or “strongly disagree” to each statement was calculated. Continuous variables were expressed as mean (± standard deviation). Categorical variables were summarised as frequencies and percentages. Comparisons were made using independent sample t-tests between (i) male and female participants; (ii) participants aged ≤ 65 years and aged > 65 years; and (iii) those prescribed insulin and those not prescribed insulin. All statistical tests were two-tailed with a P-value of 0.05 defining statistical significance. All analyses were conducted using SPSS version 22.

## Results

The questionnaire was answered by 125 people with type 2 diabetes. The mean age of participants was 65.7 ± 12.4 years and 59.2% were male. Baseline characteristics of participants are given in Table 2.

**Table 2:** Demographics and medication profile of study participants

Variable	Total population (n = 125)
Age (years)	65.7 ± 12.4
Male / female	74 (59.2) / 51 (40.8)
Oral antihyperglycaemic medicine	123 (98.4)
Parenteral antihyperglycaemic medicine	15 (12.0)
Insulin	20 (16.0)
Anti-hypertensive medication	100 (80.0)
Cholesterol-lowering medication	100 (80.0)

Continuous data are presented as mean (± standard deviation). Categorical data presented as number (%)

All but two participants were prescribed oral antihyperglycaemic therapy (98.4%). Parenteral blood glucose lowering agents were prescribed to 12.0% of participants and insulin was prescribed to 16.0% of participants. Antihypertensive and lipid-lowering therapies were each prescribed to 80.0% of patients; and 66.8% of patients were prescribed concomitant antihypertensive and lipid-lowering therapy.

The statement that participants most strongly agreed with was statement 1 “*I am totally satisfied with my visit to this pharmacist”* (combined agree or strongly agree = 99.2%, mean score 4.9 ± 0.4). Strong agreement was also expressed with statement 2 *“This pharmacist told me everything about my treatment”* (combined agree or strongly agree = 96.8%, mean score 4.7 ± 0.6) and statement 13 *“I would recommend seeing a pharmacist to other people”* (combined agree or strongly agree = 95.2%, mean score 4.7 ± 0.7).

A large number of participants (81.6%) agreed or strongly agreed with statement 9 *“It is easier to get to see the pharmacist than the doctor”* (mean score 4.3 ± 1.1) whereas just 42.4% agreed or strongly agreed with statement 11 *“I am more comfortable discussing medication-related issues with the pharmacist than my doctor”* (mean score 3.5 ± 1.2). Just 8.8% of participants agreed or strongly agreed with statement 3 *“Some things about my consultation with the pharmacist could have been better”* (mean score 1.7 ± 1.1)*.*

**Fig. 1:**
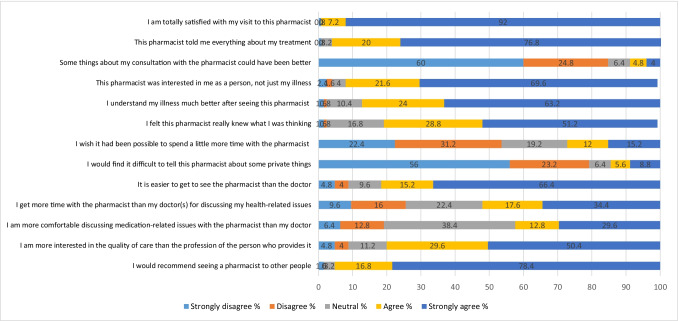
Response to each questionnaire item, expressed on a Likert scale of Strongly disagree to Strongly agree

The mean score for each questionnaire statement is given in Table 1 and the Likert scale responses of participants to each questionnaire statement are given in Fig. 1.

Female participants had higher mean scores than male participants in agreement with Statement 2 (4.88 ± 0.33 vs. 4.59 ± 0.72, p = 0.003); Statement 4 “*This pharmacist was interested in me as a person, not just my illness*” (4.78 ± 0.67 vs. 4.40 ± 0.92, p = 0.008) and Statement 5 “*I understand my illness much better after seeing this pharmacist*” (4.65 ± 0.59 vs. 4.34 ± 0.95, p = 0.028). Older participants had higher mean scores compared to younger participants in agreement with Statement 6 “*I felt this pharmacist really knew what I was thinking*” (4.42 ± 0.79 vs. 4.08 ± 0.99, p = 0.037). Participants who were prescribed insulin had higher mean scores than non-insulin prescribed participants in agreement with Statement 1 (5.00 ± 0.0 vs. 4.88 ± 0.47, p = 0.018).

## Discussion

In this study of 125 people with type 2 diabetes, it was found that 99% were fully satisfied with their visit to their community pharmacist. Over 95% agreed that the pharmacist had told them everything about their medicines and would recommend the pharmacist to others. While many participants were more comfortable discussing their medicines with their doctor, the majority of participants felt that it was easier to see a pharmacist than to access their doctor.

In this study, people with type 2 diabetes demonstrated a high level of satisfaction with their community pharmacist. This is similar to the findings of Stewart et al. who in a larger sample in England and Scotland, found that 99% agreed that they were satisfied with the prescribing service provided by their pharmacist [16]. A high level of satisfaction with the pharmacist services shows the value placed by patients on the skills and knowledge of the pharmacy profession and its translation into excellence in patient care.

Over 80% of participants agreed that it is easier to access the pharmacist than the doctor. According to Tsuyuki et al., patients have a higher level of interaction with community pharmacists than with their general practitioner and may visit a community pharmacy up to 10 times more often than they visit their general practitioner [17]. Ease of access to the community pharmacy can be utilized in the future by health services. Many health services, including in Ireland, are experiencing an ongoing shortfall in general practitioners [18]. Pharmacists are ideally placed to provide enhanced services to people with chronic illness including type 2 diabetes.

Most participants were prescribed antihypertensive and lipid lowering medications. The high risk of cardiovascular disease among people with type 2 diabetes is well documented. A sub-study of the RxEACH trial demonstrated that a pharmacist intervention among people with diabetes was associated with a significant improvement in participants’ cardiovascular risk and an improvement in individual cardiovascular risk factors when compared to usual pharmacy care [6]. The challenge for pharmacists in Ireland is translating effective clinical trial interventions into everyday clinical practice and achieving reimbursement for this activity.

Some participants were more comfortable discussing their medications with their doctor than with their pharmacist. This result reflects the findings of a qualitative study conducted by Twigg et al. in the UK concerning current and future roles of community pharmacists and the views and experiences of people with type 2 diabetes [19]. In their study, Twigg et al. found that people with type 2 diabetes placed an emphasis on the pharmacist’s specific role in supply of medicines and the pharmacist’s knowledge of over-the-counter medicines but were less clear on the pharmacist’s role in providing advice on prescription medicines [19]. This finding aligns with the present study, where “*I am more comfortable discussing medication-related issues with the pharmacist than my doctor*” was one of the lower-scoring statements. However, a relatively high percentage of participants gave a neutral response to this question. The wording of the question may have influenced participants who answered neutrally, and the authors cannot say how participants may have responded had they been asked if they are “*as comfortable*”, rather than “*more comfortable*” discussing medication-related issues with their pharmacist than with their doctor. The responses to this statement demonstrate the importance of broadening patient understanding of the knowledge and skills-base of community pharmacists in the management of chronic disease.

Conducting the study on site in the pharmacy and in the presence of the researcher may have influenced participant responses. Although consecutive people with type 2 diabetes attending each pharmacy were approached about the study, not all consented. As the number of people with diabetes approached about the study was not collected, the response rate could therefore not be calculated. As the participants were self-selecting, some respondents may have had a predisposed positive attitude towards pharmacist care which may influence the results. The aim of the study was to evaluate the views and experiences of people with type 2 diabetes attending their pharmacy, and so the questionnaire was not validated against any clinical outcomes and a sample size calculation was not performed. Therefore, neither a precision margin nor 95% confidence interval could be calculated. Data on time since diagnosis of type 2 diabetes or duration of time attending the study pharmacy was not collected. It is possible that these factors may have influenced the views of participants towards their community pharmacist.

Future research may focus on pharmacists’ views on delivering advanced care to people with type 2 diabetes and a baseline evaluation of patient needs for developing a pharmacist-led intervention in type 2 diabetes in Ireland.

## Conclusion

In this population, people with type 2 diabetes were highly satisfied with the care provided to them by their community pharmacist. These data support the implementation of enhanced community pharmacy services for people with type 2 diabetes in Ireland.

## Data Availability

The datasets generated and analysed during the current study are available from the corresponding author on reasonable request.
